# Does deep learning software improve the consistency and performance of radiologists with various levels of experience in assessing bi-parametric prostate MRI?

**DOI:** 10.1186/s13244-023-01386-w

**Published:** 2023-03-20

**Authors:** Aydan Arslan, Deniz Alis, Servet Erdemli, Mustafa Ege Seker, Gokberk Zeybel, Sabri Sirolu, Serpil Kurtcan, Ercan Karaarslan

**Affiliations:** 1 Department of Radiology, Umraniye Training and Research Hospital, Istanbul, Turkey; 2 Department of Radiology, School of Medicine, Acibadem Mehmet Ali Aydinlar University, Istanbul, Turkey; 3 School of Medicine, Acibadem Mehmet Ali Aydinlar University, Istanbul, Turkey; 4 Department of Radiology, Istanbul Sisli Hamidiye Etfal Training and Research Hospital, Istanbul, Turkey; 5 Department of Radiology, Acibadem Healthcare Group, Istanbul, Turkey

**Keywords:** Deep learning, Magnetic resonance imaging, Prostate cancer

## Abstract

**Objective:**

To investigate whether commercially available deep learning (DL) software improves the Prostate Imaging-Reporting and Data System (PI-RADS) scoring consistency on bi-parametric MRI among radiologists with various levels of experience; to assess whether the DL software improves the performance of the radiologists in identifying clinically significant prostate cancer (csPCa).

**Methods:**

We retrospectively enrolled consecutive men who underwent bi-parametric prostate MRI at a 3 T scanner due to suspicion of PCa. Four radiologists with 2, 3, 5, and > 20 years of experience evaluated the bi-parametric prostate MRI scans with and without the DL software. Whole-mount pathology or MRI/ultrasound fusion-guided biopsy was the reference. The area under the receiver operating curve (AUROC) was calculated for each radiologist with and without the DL software and compared using De Long’s test. In addition, the inter-rater agreement was investigated using kappa statistics.

**Results:**

In all, 153 men with a mean age of 63.59 ± 7.56 years (range 53–80) were enrolled in the study. In the study sample, 45 men (29.80%) had clinically significant PCa. During the reading with the DL software, the radiologists changed their initial scores in 1/153 (0.65%), 2/153 (1.3%), 0/153 (0%), and 3/153 (1.9%) of the patients, yielding no significant increase in the AUROC (*p* > 0.05). Fleiss’ kappa scores among the radiologists were 0.39 and 0.40 with and without the DL software (*p* = 0.56).

**Conclusions:**

The commercially available DL software does not increase the consistency of the bi-parametric PI-RADS scoring or csPCa detection performance of radiologists with varying levels of experience.

## Key points

- Radiologists with varying experiences assigned the scores with and without the DL.
- The radiologists infrequently changed their initial PI-RADS scores.
- The DL software did not improve the PI-RADS scoring consistency.
- The DL software did not improve the performance in identifying csPCa.

## Introduction

Magnetic resonance imaging (MRI) is the backbone imaging modality for assessing prostate cancer (PCa). The role of MRI in evaluating PCa has recently heightened as the new evidence suggests the benefits of pre-biopsy MRI in all men with a suspicion of PCa [1–3]. As the importance of MRI in PCa diagnosis increased, the Prostate Imaging-Reporting and Data System (PI-RADS) and its following versions were introduced to bring standardization [4]. The PI-RADS provides guidelines for acquiring and interpreting prostate MRI, and the benefits of the system have been demonstrated in large-scale multi-center studies [5]. However, despite the PI-RADS, there are still non-negligible intra-reader and inter-reader differences in interpreting prostate MRI [6]. Furthermore, the inconsistencies appear to be more prominent with the less-experienced readers, hindering the standardization efforts [6].

Deep learning (DL) has shown remarkable performance on prostate MRI in recent years, including PCa detection, classification, and segmentation [7–9]. Nevertheless, a few studies have explicitly investigated whether DL benefits in standardizing the PI-RADS scoring among radiologists [10–12]. Further, prior studies have used in-house algorithms or prototype DL software; hence, there is a need for evidence for the yields of regulatory body-approved commercially available DL software in standardizing the PI-RADS scores and improving the performance of radiologists in identifying clinically significant PCa (csPCa).

The aims of this study were twofold: First, to investigate whether the commercially available DL software increases the PI-RADS scoring consistency on bi-parametric MRI among radiologists with various experience levels; Second, to assess whether the DL software improves the performance of radiologists in identifying csPCa.

## Methods

Acibadem University Review board approved this retrospective study (ID: 2022-05/08) and waived the need for informed consent for the retrospective analysis of anonymized medical data. We reviewed consecutive patients who underwent a prostate MRI scan due to suspicion of PCa (i.e., increased prostate-specific antigen or suspicious digital rectal examination) or active surveillance between January 2019 and December 2020.

The inclusion criteria were the followings: (1) having whole-mount pathology or biopsy for patients with a PI-RADS ≥ 3 score assigned during routine clinical reading; (2) having a prostate MRI scan obtained at 3 T without an endorectal coil following PI-RADS version 2; and (3) ≥ 18 months of follow-up without any clinical, laboratory, or imaging evidence of PCa for patients with a PI-RADS score ≤ 2 [13].

The following patients were excluded from the study: (1) patients who underwent prostate MRI at 1.5 T; (2) patients who underwent prostate MRI with an endorectal coil; (3) patients with PI-RADS ≥ 3 examinations without any histopathological confirmation; and (4) history of any treatment for PCa.

### MRI acquisitions

All patients underwent prostate MRI on one of our 3 Tesla MRI units (Vida or Skyra, Siemens Healthcare) using an 18-channel phased-array surface coil. The MRI protocol was consistent with PI-RADS version 2, as version 2.1 was unavailable during the study period [4]. To minimize bowel movements, Butylscopolamine bromide (Buscopan, Bohringer Ingelheim) was given to the patients.

The bi-parametric prostate MRI protocol encompassed tri-planar T2-weighted imaging and diffusion-weighted imaging. The diffusion-weighted imaging was performed with echo-planar imaging in axial planes at *b*-values of 0, 50, 500, and 1000 s/mm^2^. We excluded dynamic contrast-enhanced images since the DL software could not process them. The detailed parameters of the MRI protocol are given in Table 1.

**Table 1:** The detailed prostate multiparametric magnetic resonance imaging parameters

Parameters	Turbo spin-echo T2-weighted imaging			Diffusion-weighted imaging
Plane	Axial	Sagittal	Coronal	Axial
Time-to-repeat (ms)	5500	5040	3900	4800
Time-to-echo (ms)	104	115	117	63
Time of acquisition (min)	3.21	2.33	3.05	4.02
Field-of-view (mm)	200	220	240	200
Slice thickness (mm)	3	3	3	3
*b*-values (s/mm^2^)	–	–	–	0, 50, 500, 1000
Matrix size	384 × 307	384 × 288	448 × 291	114 × 88

### DL software

The DL software (Prostate AI, Version Syngo.Via VB60, Siemens Healthcare) used in this study has three modules: (i) preprocessing module, (ii) DL-based lesion detection module, and (iii) DL-based lesion classification module. In this study, we did not perform any model training or fine-tuning and only used the model for performance testing.

#### Preprocessing module

The preprocessing module parses the DICOM files to select the axial T2-weighted and DWI with various *b*-values (e.g., 0 s/mm^2^ and 800 s/mm^2^). Then, the preprocessing module computes the ADC maps and synthetic DWI with a *b*-value of 2000 s/mm^2^ using a linear least-square fitting with all acquired *b*-values (i.e., *b*-values of 0, 50, 500, and 1000 s/mm^2^ for this study). Afterward, it performs prostate segmentation on T2-weighted images using a DL method proposed by Yang et al. [14] and rigid registration of T2-weighted and DWI.

#### DL-based lesion detection module

Preprocessed images are propagated into the DL-based lesion detection module. This module has two subcomponents: (1) DL-based lesion candidate detection model and (2) multi-scale false-positive reduction network.

DL-based lesion candidate detection model is a simple 2D U-Net consisting of descending and ascending pathways inter-connected with skip connections at different levels and convolutional blocks at the bottom, resembling a U shape. This model takes 3D volumes of ADC, DWI with a *b*-value of 2000 s/mm^2^, and T2-weighted images but processes them slice by slice. The model outputs 2D heatmaps fused to create 3D connected components (i.e., lesion candidates). The detected lesion candidates then propagated into the false-positive reduction model.

The false-positive reduction model is a 2.5D multi-scale deep network previously trained and validated on radiologists-annotated 2170 bi-parametric prostate MRI scans from 7 institutions. The model takes the patches of ADC, DWI, and T2-weighted images of lesion candidates provided by the DL-based lesion candidate detection model.

A 2D DL model can assess the in-plane information within an image (i.e., x and y axes), while it cannot capture the out-of-plane information (i.e., z-axis). Given that the prostate images contain relevant information in the x, y, and z axes, it is essential to consider the information of all planes in evaluating prostate MRI, particularly for eliminating false-positive lesions. Hence, the false-positive reduction model takes two adjacent slices of a 2D input slice as additional channels, making it a 2.5D network. For instance, a T2-weighted image harboring a lesion is fed to the model along with a slice above and below it. This design allows the network to capture the information z-axis and improves consistency and performance. At the same time, it mitigates the need for using fully 3D DL networks, which are resource intensive. In addition, this model is fed by prostate images with a varying field of view (i.e., multi-scale) to empower the model in capturing additional contextual information.

#### DL-based lesion classification module

The final module of the DL software is the lesion classification module. This module takes the lesion candidates offered by the preceding lesion detection module and provides the PI-RADS scores of the lesion, if present, as PI-RADS 3, 4, or 5, and highlights the lesions on the axial T2-weighted images. Supplementary Document S1 illustrates the components of the DL software. A further detailed description of the DL software can be found in Yu et al. [15].

### Radiologists reading

Four radiologists with varying experience levels interpreted the scans with and without the DL software on a dedicated workstation (Syngo.Via, Siemens Healthcare) equipped with a 6-megapixel diagnostic color monitor (Radiforce RX 660, EIZO). All reviewed images were in Digital Imaging and Communications in Medicine (DICOM) format. The first reader was a radiologist with > 20 years of experience. The remaining three radiologists had 5, 3, and 2 years of prostate MRI experience and were routinely interpreting less than 50 prostate MRI scans yearly (hereafter, these radiologists were denoted as less-experienced radiologists 1, 2, and 3, respectively). All radiologists were briefly instructed about the software before the reading.

The radiologists evaluated the scans following PI-RADS version 2, as the DL software used in this study was developed following PI-RADS version 2. With multiparametric prostate MRI, PI-RADS 3 lesions of the peripheral zone showing focal or early contrast-enhancement are upgraded to PI-RADS 4 (i.e., PI-RADS 3 + 1) following PI-RADS version 2 [4]. However, as the contrast-enhanced sequences are not available in bi-parametric MRI, lesions of the peripheral gland are scored using only the diffusion-weighted sequences. Thus, in this study, none of the PI-RADS 3 lesions of the peripheral zone were upgraded to a higher score.

In the initial readings, the radiologists were provided with bi-parametric MRI scans including high *b*-value DWI and asked to identify the index lesion (i.e., the lesion with the highest PI-RADS score or the largest lesion if there were ≥ 2 lesions with the same score). First, the radiologists marked the index lesion with its PI-RADS score using the standard prostate reading template [4]. Then the radiologists were provided with the decision of DL software overlaid on a T2-weighted image and asked to re-evaluate the scans to assess whether they changed their initial PI-RADS score. Likewise, the PI-RADS scores of the radiologists with the DL software were recorded in the same template. Supplementary Document S2 shows how radiologists read the cases with and without the DL software step by step.

### Whole-mount histopathology and biopsy

All biopsy procedures involved a combination of transrectal 12-core systematic and 3–4-core MRI/ultrasound fusion-guided biopsies (Artemis, Eigen) following up-to-date evidence [16]. Biopsy and whole-mount specimens were prepared and evaluated by a genitourinary pathologist with over 20 years of experience following international guidelines [16]. The lesion with the highest Gleason score was defined as the index lesion. A lesion with a Gleason score ≥ 3 + 4 was defined as a clinically significant PCa following the 2014 International Society of Urological Pathology consensus [17].

### Statistical analysis

The statistical analyses were performed using the SciPy library of the Python programming language. The continuous variables are presented using the mean and standard deviations with the minimum and maximum; the categorical and ordinal variables are presented with frequencies and percentages. The PI-RADS scores of the radiologists were calculated and compared on a scan level. The inter-rater agreement among the radiologists in PI-RADS scoring with and without the DL software was evaluated using Fleiss’ kappa [18]; the pair-wise inter-rater agreements were investigated using linearly weighted Cohen’s kappa [19]. The kappa scores were interpreted as follows: a kappa score of < 20, a poor agreement; 21–40, a fair agreement; 41–60, a moderate agreement; 61–80, a good agreement; and 81–100, an excellent agreement. The kappa scores were compared following the prior work [20]. We calculated the area under the receiver operating curve (AUROC) in assessing csPCa and compared the AUROCs using DeLong’s test. A *p* value less than 0.05 was accepted as significant.

## Results

In all, 153 men with a mean age of 63.59 ± 7.56 years (range 53–80) were enrolled in the study. The mean prostate-specific antigen level of the men was 6.42 ± 3.87 ng/ml (range 2–24).

**Fig. 1:**
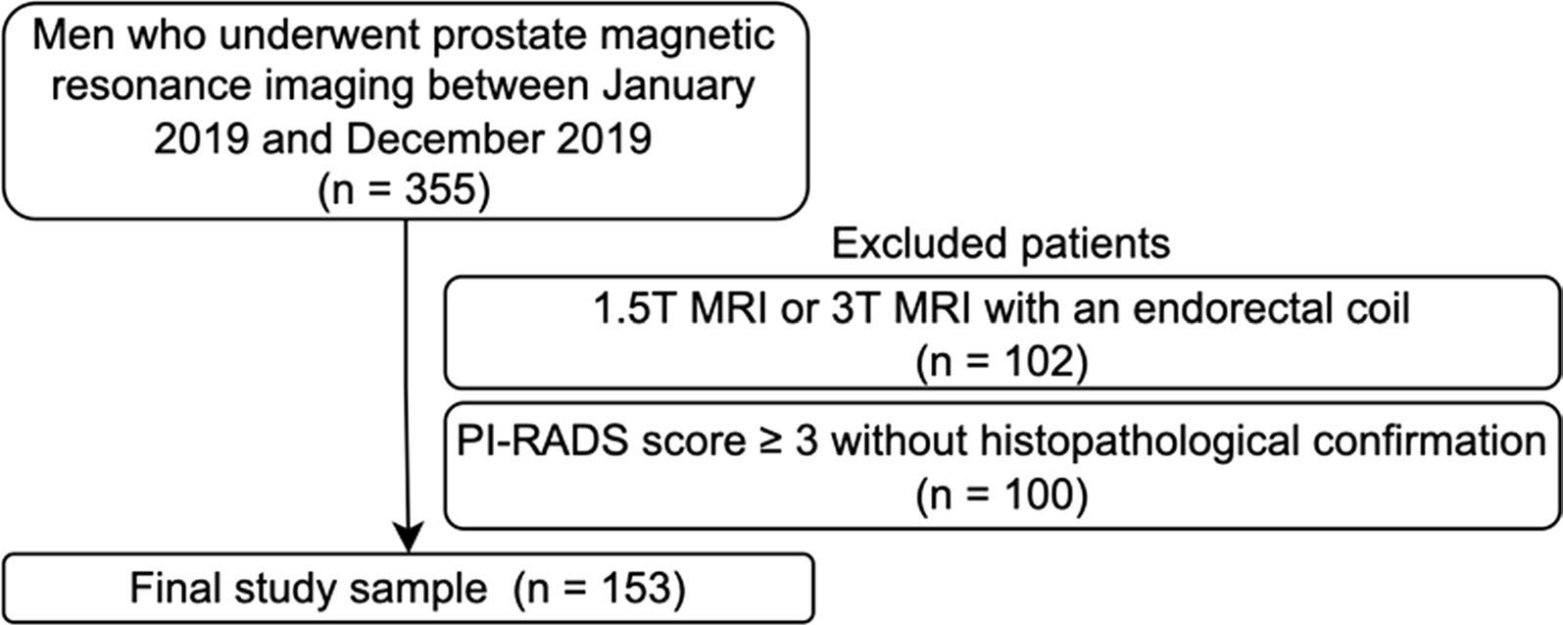
The flowchart of the study

Of 153 men, 113 (75.16%) had a histopathology result, with 45 (29.41%) having clinically significant PCa identified by whole-mount pathology superseding the biopsy, 31 (20.26%) had nonsignificant PCa identified by whole-mount pathology (*n* = 5, 3.26%) or biopsy (*n* = 26, 16.99%), and 39 (25.49%) had a benign disease as identified by biopsy. The remaining 38 men (24.83%) had a PI-RADS score of ≤ 2 on MRI and ≥ 18 months of follow-up without any clinical, laboratory, or imaging evidence of PCa (Fig. 1).

### The inter-rater agreement among the radiologist with and without the DL software

**Fig. 2:**
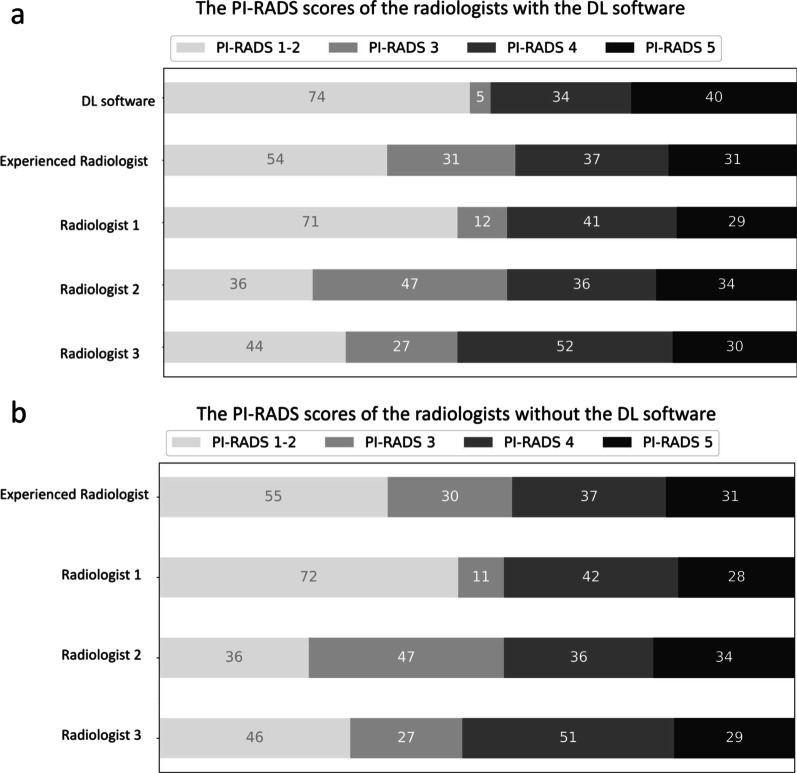
Horizontal bar charts show the PI-RADS scores assigned by the radiologists with (**a**) and without (**b**) the DL software

The PI-RADS scores assigned by the radiologists with and without DL are given in Fig. 2. Notably, the radiologists changed their initial PI-RADS scores in 1/153 (0.65%), 2/153 (1.3%), 0/153 (0%), and 3/153 (1.9%) of the patients with the DL software.

**Fig. 3:**
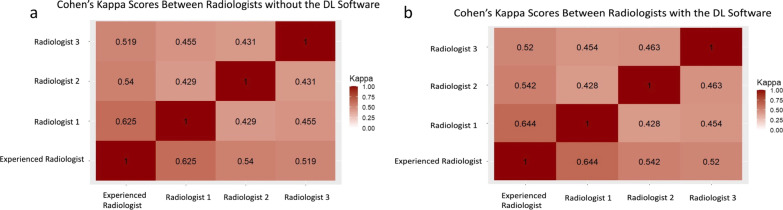
Cohen’s kappa scores between radiologists without (**a**) and with (**b**) the software. There was no statistical difference between the pair-wise kappa scores with and without the DL software

**Fig. 4:**
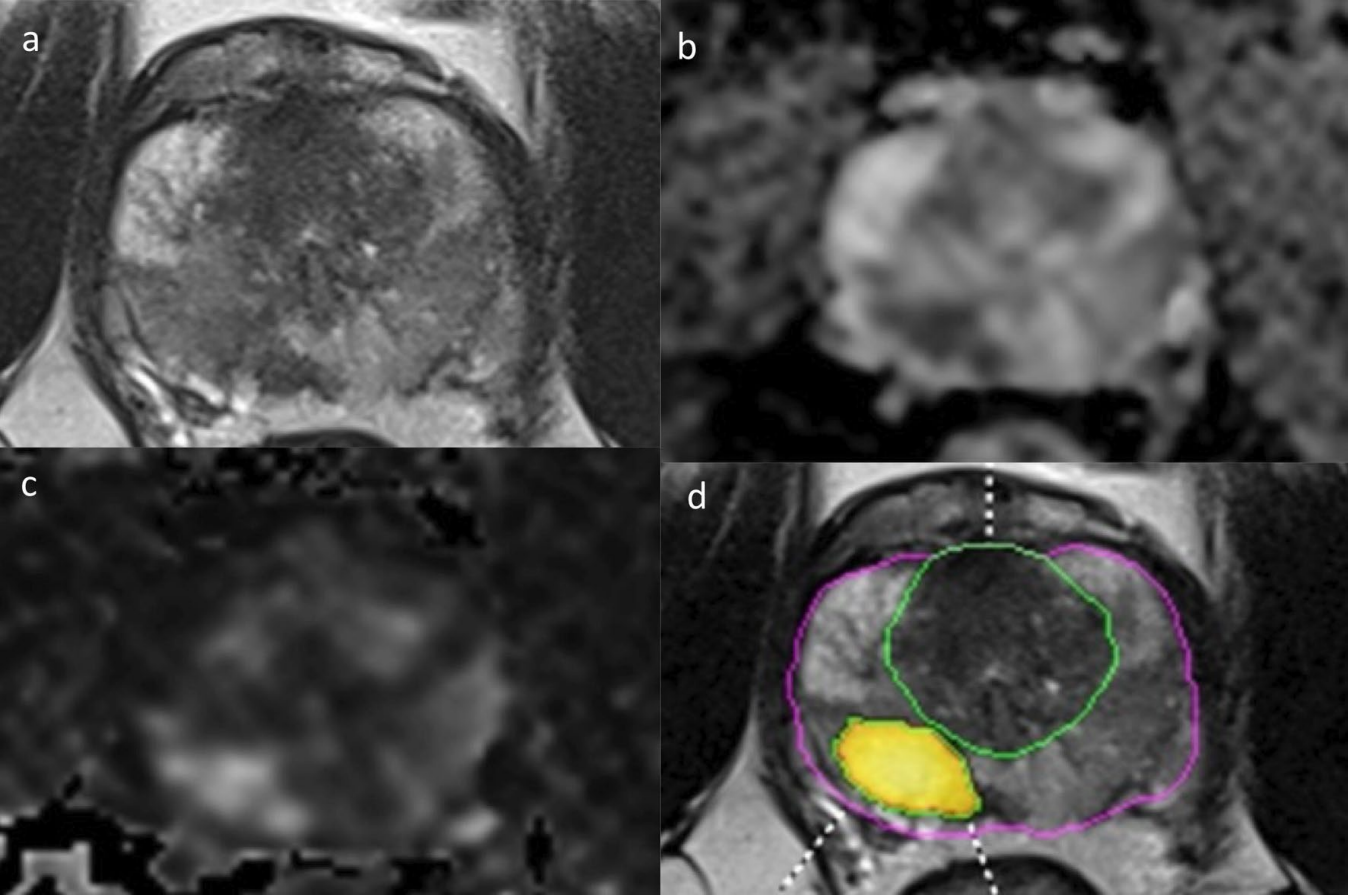
Radiologists and the DL software in assigning PI-RADS scores. A 64-year-old man with prostate adenocarcinoma with a Gleason Score of 4 + 3 in the right posterolateral peripheral gland at the mid-prostatic level. An axial T2-weighted imaging (**a**), apparent diffusion coefficient map (**b**), diffusion-weighted imaging with a high *b*-value (**c**), and deep learning decisions overlaid on T2-weighted imaging with a heatmap (**d**) are shown. The radiologists scored this lesion as PI-RADS 5 with and without the DL software

**Fig. 5:**
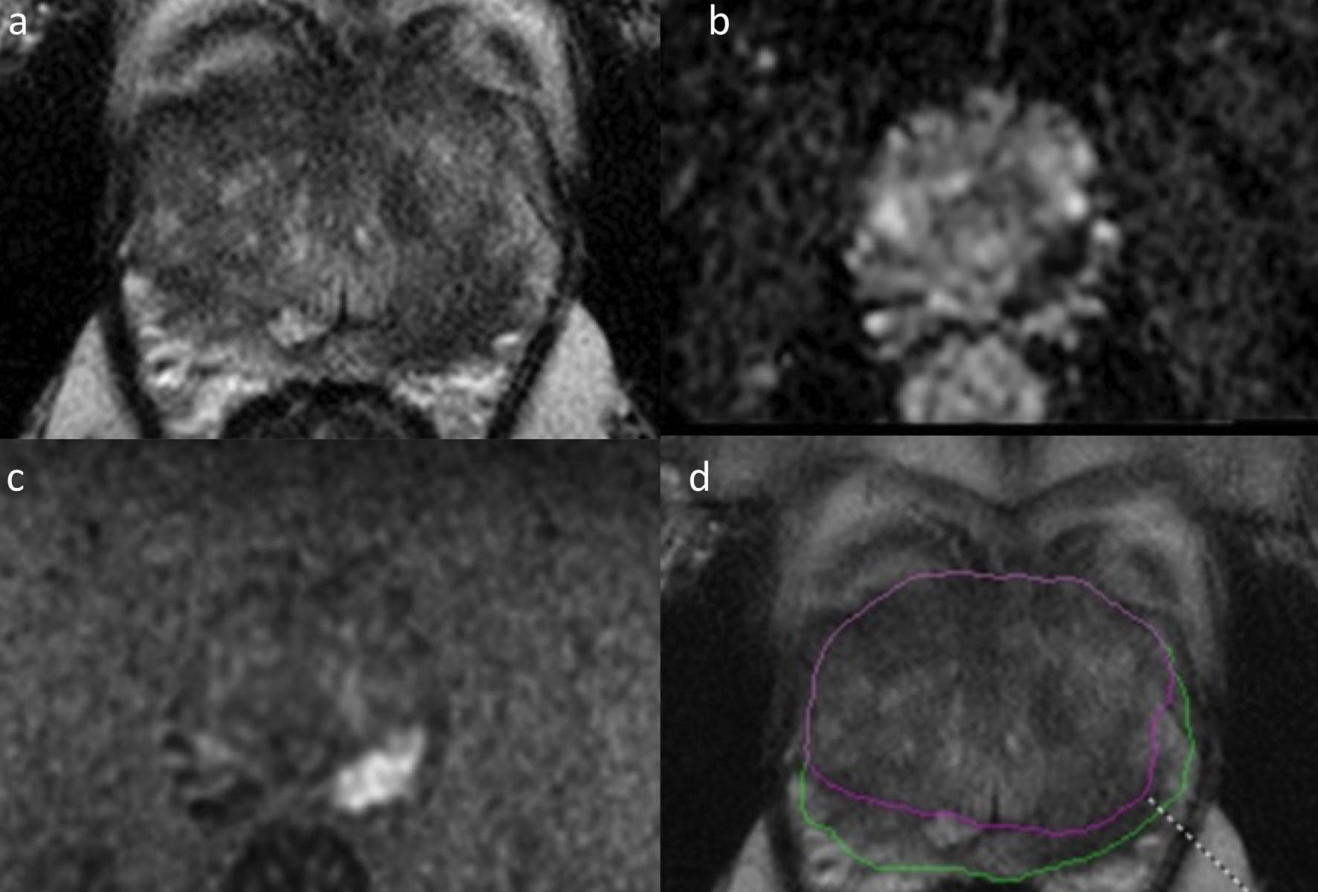
Radiologists and the DL software in assigning PI-RADS scores. A 55-year-old man with clinically significant prostate adenocarcinoma with a Gleason Score of 4 + 3 in the left posterior peripheral gland at the basal level. An axial T2-weighted imaging (**a**), apparent diffusion coefficient map (**b**), diffusion-weighted imaging with a high *b*-value (**c**), and deep learning decisions overlaid on T2-weighted imaging with a heatmap (**d**) are shown. All radiologists scored PI-RADS 5 for the index lesion. However, the deep learning software failed to identify the index lesion

Fleiss’ kappa Score among the radiologists without the DL software was 0.39, equating to a fair agreement. Fleiss’ kappa Score among the radiologists increased from 0.39 to 0.40 with the DL software, not representing a significant difference (*p* = 0.56). The pair-wise kappa scores among radiologists with and without the DL software are shown in Fig. 3. Figures 4 and 5 show representative patients with clinically significant PCa.

### The performance of the radiologists in identifying csPCa with and without DL software

**Fig. 6:**
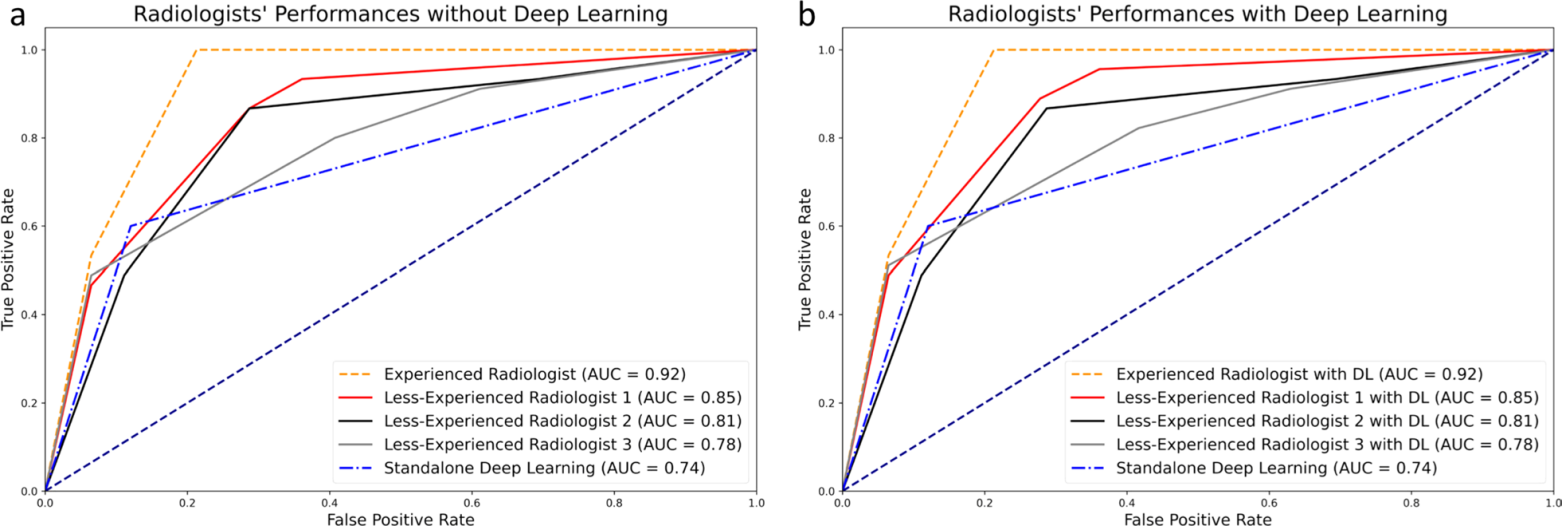
The area under the receiver operating curves. The area under the receiver operating curves of the radiologists without (**a**) and with (**b**) the deep learning software in identifying clinically significant prostate cancer

The AUROCs of the experienced radiologist, less-experienced radiologist 1, less-experienced radiologist 2, and less-experienced radiologist 3 without the DL software were 0.917 (95% CI 0.878–0.957), 0.847 (95% CI 0.785–0.909), 0.81 (95% CI 0.733–0.883), 0.782 (95% CI 0.702–0.862). The AUROC of the standalone DL software was 0.756 (95% CI 0.671–0.842). The AUROCs of the experienced radiologist, less-experienced radiologist 1, less-experienced radiologist 2, and less-experienced radiologist 3 with the DL software were 0.917 (95% CI 0.878–0.957), 0.864 (95% CI 0.806–0.921), 0.81 (95% CI 0.733–0.883), and 0.789 (95% CI 0.710–0.868). Figure 6 shows the ROC curves of the radiologists with and without the DL software in predicting clinically significant PCa.

The AUROCs of the experienced radiologist and less-experienced radiologist 1 were significantly higher than that of the DL software (*p* < 0.0001 and *p* = 0.04). In contrast, the AUROCs of the remaining less-experienced radiologists 2 and 3 did not significantly differ from that of the DL software (*p* = 0.63 and *p* = 0.23). The AUROCs of radiologists in identifying csPCa with and without the DL software did not differ for radiologists (*p* > 0.05).

## Discussion

This study investigated whether DL improves the consistency and performance of radiologists with various levels of experience in assessing bi-parametric prostate MRI. In this study, there was a fair agreement between the radiologists in assigning the bi-parametric PI-RADS scores, and the inter-rater agreement among radiologists did not significantly increase using the DL software. Overall, the radiologists changed their initial PI-RADS scores in ~ 1% of the scans with the DL software, and radiologists with ≥ 5 years of experience provided a statistically higher performance in identifying csPCa than the DL software. Furthermore, the DL software did not improve radiologists’ performance in identifying csPCa.

In this study, the DL software used PI-RADS 3 sparingly while mainly allocating scans as negative or highly suspicious of cancer (i.e., PI-RADS score of ≥ 4). A similar trend was also reported by prior research using the prototype version of the DL software [10, 21]. In contrast, the experienced radiologist assigned a PI-RADS score of 3 to about a quarter of the patients in the present work. Likewise, a recent meta-analysis pooled data across 26 centers showed that approximately 30% of the lesions were assigned a PI-RADS score of 3 by radiologists [5]. We suggest that the potential underlying factors that lead the DL software to assign PI-RADS 3 to only a minority of the patients and whether this tendency is beneficial (e.g., sparing patients from unnecessary biopsy or identifying clinically insignificant cancers) should be investigated in future work.

The experienced radiologist provided an AUROC of 0.917 in identifying significant PCa, compatible with the literature [5, 21]. On the other hand, standalone DL software had a worse performance, with an AUROC of 0.756 in the same task. Furthermore, the radiologists with 5 years of experience also performed better than the DL software, while the radiologist with 3 and 2 years of experience provided a similar performance. These findings might imply that the DL software used in this study might be at the same level as a radiologist with ≤ 3 years of experience, while it fails to match the performance of radiologists with more experience.

At first glance, the low performance of DL software appears to contradict the results from the earlier studies using the prototype version of the same DL software [11, 21]. However, prior studies tested the DL software on in-distribution or ProstateX data containing relatively straightforward cases. Though our MRI scans were obtained with the same manufacturer’s scanner, it might represent out-of-distribution data for the DL software, eventually impairing its performance. Furthermore, our findings, to some extent, align with those from the study by Youn et al., where the authors found that the prototype version of the DL software had a performance between an expert radiologist and radiology residents [21].

Many recent studies have claimed that DL could surpass human radiologists in identifying clinically significant PCa [22–26]. Nevertheless, in a recent large-scale multi-center study, Hosseinzadeh criticized earlier studies and suggested that small test sizes and the comparison with local radiologists’ performance might lead to overestimating the performance of DL [27]. The authors stated that those prostate DL models trained less than 1 k scans and pointed out that a DL model with an expert-level performance could only be achieved by training over ≥ 35 k scans for lung cancer detection on CT and 90 ≥ scans for breast cancer detection on mammography [28, 29]. However, the commercial DL software used in this study trained slightly over 2 k scans [10].

Considering the current DL models that were trained on relatively small data, expecting an expert-level performance might be too optimistic at this moment. Further, despite its drawbacks regarding consistency, the benefits of the PI-RADS scores assigned by human radiologists are much more rigorously documented than DL in PCa diagnostics [5]. Thus, we suggest that creating a DL software that can replace human radiologists might be a longer-term goal, while designing DL models that improve and standardize the PI-RADS scores among radiologists seems to be a more reachable target [30].

Apart from the present work, few other studies specifically investigated the benefits of DL in the context of PI-RADS scoring consistency. For example, in their large-scale study, Sanford et al. evaluated the inter-rater agreement between their in-house deep learning model, U-net, and human experts [12]. The authors documented a moderate level of agreement between the software and experts. However, unlike the present work, the authors did not investigate whether prostate MRI reading with the DL model improves the inter-rater agreement.

Winkel et al. investigated the prototype version of the DL software used in the present work in two consecutive studies [10, 11]. Their preliminary study was small-scale and only examined the inter-rater agreement between radiology reports and DL software, yielding a kappa score of 0.42. Their subsequent study used a similar methodology to the present work, and radiologists with various levels of experience performed prostate MRI readings with and without the DL software. In contrast to the present work, the DL software improved the inter-rater agreement in their research. However, the authors binarized the PI-RADS scores using the cut-off values of PI-RADS 3 and 4. Using PI-RADS ≥ 3 cut-off threshold, the inter-rater agreement increased from kappa of 0.33 to 0.41 with the DL software, while it was increased from kappa of 0.22 to 0.36 using a PI-RADS ≥ 4 cut-off threshold.

This study had several drawbacks that should be acknowledged. First and foremost, the sample size was relatively small, covering prostate MRI scans obtained with the same manufacturer’s 3 T scanner from a single tertiary center.

Second, we used bi-parametric MRI since the DL software used in this study does not use dynamic contrast-enhanced images. Though the performance of bi-parametric MRI is on par with multiparametric MRI [31], various guidelines [32–34], including the PI-RADS [4], still recommend multiparametric MRI over bi-parametric prostate MRI. Thus, the results of the study might not be applicable to routine practice where the multiparametric MRI is routinely implemented for patient care. Likewise, we put PI-RADS scores 1 and 2 in the same category (i.e., negative scans) as the DL software does not discriminate between them.

Third, some patients with PI-RADS scores 1 and 2 did not have histopathology in our sample. Nevertheless, these patients had at least 18 months of follow-up data, and the same approach was also followed in the recent large-scale prostate cancer detection challenge [13].

Fourth, we only evaluated whether the DL software improves the diagnostic accuracy in detecting csPCa and the consistency of radiologists with varying experience. We admit that this might downplay the role of DL in prostate diagnostics. For instance, DL may help to reduce prostate MRI reading time, lessening the daily workload of radiologists [11, 30].

## Conclusions

In contrast to most earlier studies, the commercially available DL software did not improve the PI-RADS scoring or csPCa detection performance of radiologists with varying levels of experience on external single-center data, potentially suggesting a drop in the performance due to a domain shift. Though we suggest that prostate MRI practitioners should consider a potential drop in the performance using the version of the DL software in clinical practice, the potential benefits of the DL, such as improving efficiency, should not be overlooked. In subsequent studies, we plan to investigate the benefits of the DL software in reading efficiency and confidence, along with the accuracy and consistency of a larger pool of radiologists from different centers on large-scale multi-center data. Furthermore, advancements in the DL technology, accompanied by larger and more representative training data, will likely improve the performance of the DL software in future versions, which we plan to investigate in future studies.

## Data Availability

The datasets generated during and/or analyzed during the current study are available from the corresponding author upon reasonable request.

## Abbreviations

AUROC: The area under the receiver operating curve
csPCa: Clinically significant PCa
DL: Deep learning
MRI: Magnetic resonance imaging
PCa: Prostate cancer
PI-RADS: Prostate Imaging-Reporting and Data System
